# Assessment of Myocardial Function During Blood Pressure Manipulations Using Feature Tracking Cardiovascular Magnetic Resonance

**DOI:** 10.3389/fcvm.2021.743849

**Published:** 2021-10-12

**Authors:** Kady Fischer, Mario D. Neuenschwander, Christof Jung, Samuel Hurni, Bernhard M. Winkler, Stefan P. Huettenmoser, Bernd Jung, Andreas P. Vogt, Balthasar Eberle, Dominik P. Guensch

**Affiliations:** ^1^Department of Anaesthesiology and Pain Medicine, Inselspital, Bern University Hospital, University of Bern, Bern, Switzerland; ^2^Department of Diagnostic, Interventional and Paediatric Radiology, Inselspital, Bern University Hospital, University of Bern, Bern, Switzerland; ^3^Department of Cardiovascular Surgery, Inselspital, University Hospital Bern, Bern, Switzerland

**Keywords:** feature tracking (CMR-FT), general anesthesia (GA), blood pressure, coronary autoregulation, myocardial deformation

## Abstract

**Background:** Coronary autoregulation is a feedback system, which maintains near-constant myocardial blood flow over a range of mean arterial pressure (MAP). Yet in emergency or peri-operative situations, hypotensive or hypertensive episodes may quickly arise. It is not yet established how rapid blood pressure changes outside of the autoregulation zone (ARZ) impact left (LV) and right ventricular (RV) function. Using cardiovascular magnetic resonance (CMR) imaging, measurements of myocardial tissue oxygenation and ventricular systolic and diastolic function can comprehensively assess the heart throughout a range of changing blood pressures.

**Design and methods:** In 10 anesthetized swine, MAP was varied in steps of 10–15 mmHg from 29 to 196 mmHg using phenylephrine and urapidil inside a 3-Tesla MRI scanner. At each MAP level, oxygenation-sensitive (OS) cine images along with arterial and coronary sinus blood gas samples were obtained and blood flow was measured from a surgically implanted flow probe on the left anterior descending coronary artery. Using CMR feature tracking-software, LV and RV circumferential systolic and diastolic strain parameters were measured from the myocardial oxygenation cines.

**Results:** LV and RV peak strain are compromised both below the lower limit (LV: Δ1.2 ± 0.4%, RV: Δ4.4 ± 1.2%, *p* < 0.001) and above the upper limit (LV: Δ2.1 ± 0.4, RV: Δ5.4 ± 1.4, *p* < 0.001) of the ARZ in comparison to a baseline of 70 mmHg. LV strain demonstrates a non-linear relationship with invasive and non-invasive measures of oxygenation. Specifically for the LV at hypotensive levels below the ARZ, systolic dysfunction is related to myocardial deoxygenation (β = −0.216, *p* = 0.036) in OS-CMR and both systolic and diastolic dysfunction are linked to reduced coronary blood flow (peak strain: β = −0.028, *p* = 0.047, early diastolic strain rate: β = 0.026, *p* = 0.002). These relationships were not observed at hypertensive levels.

**Conclusion:** In an animal model, biventricular function is compromised outside the coronary autoregulatory zone. Dysfunction at pressures below the lower limit is likely caused by insufficient blood flow and tissue deoxygenation. Conversely, hypertension-induced systolic and diastolic dysfunction points to high afterload as a cause. These findings from an experimental model are translatable to the clinical peri-operative environment in which myocardial deformation may have the potential to guide blood pressure management, in particular at varying individual autoregulation thresholds.

## Introduction

Coronary autoregulation ensures near constant coronary blood flow (CBF) over a wide range of blood pressure, known as the autoregulatory zone (ARZ). The general relationship of mean arterial pressure (MAP) to CBF has been well-established (1). In healthy individuals, coronary autoregulation is usually effective between MAP levels from 60 to 140 mmHg. Above and below those thresholds of the ARZ, blood flow becomes mostly pressure dependent. Thus, myocardium may be vulnerable to insufficient perfusion with subsequent ischemia at low pressures, while higher aortic pressures may increase myocardial workload and oxygen demand. Peri-operatively, even short periods of hypotension and hypertension, i.e., markedly fluctuating blood pressures, are associated with poor outcome (2, 3). However, the upper and lower limits of this ARZ differs among individuals and setting. Consequently, the exact characteristics of autoregulation in cardiovascular disease during general anesthesia remain a black box for anesthetists. This may contribute to myocardial injury after non-cardiac surgery (MINS) (4), which has been increasingly focused on over the recent years. Techniques such as oxygenation-sensitive cardiovascular magnetic resonance (OS-CMR) can non-invasively interrogate changes in myocardial oxygenation on a tissue level with high spatial resolution based on local deoxyhemoglobin fractions (5, 6). In 2019, we reported that left ventricular myocardial oxygenation measured by OS-CMR had a curvi-linear association with mean arterial pressure (7). However, the impact of blood pressure regulation and these oxygenation changes on biventricular myocardial function defined by strain have not been systematically examined so far.

Myocardial strain analysis allows for a more detailed quantitative analysis of myocardial deformation and performance on a global and segmental level. Diagnostically, deficiencies in resting CMR-FT of both the left and right ventricle has been shown to be linked to outcome in multiple ischemic and non-ischemic cohorts (8–10). In these studies, strain analysis showed more subtle abnormalities in the absence of global systolic dysfunction and is thus interpreted to be an earlier and more sensitive prognostic modality than conventional assessment of global function alone. Although strain analysis has been performed in echocardiography for many years, the use of feature tracking (FT) software on OS-CMR cine images simultaneously with oxygenation assessments is only possible with CMR. Various markers can be derived from strain analysis, of which peak strain is the most known and best validated (11), representing maximal systolic shortening. Systolic strain rate provides another marker for systolic function. Importantly, diastology can be simultaneously assessed using diastolic strain rate during early active relaxation of the myocardium. This multiparametric approach of strain assessments is beneficial as it provides a comprehensive assessment of various components of myocardial function and layers over the cardiac cycle.

The objective of this analysis was to implement advanced assessments of myocardial deformation by CMR-FT to investigate the impact of systemic mean arterial blood pressure changes on left and right ventricular strain in an anesthetized swine model. Additionally, we aimed to establish the relationship of biventricular strain with changes in myocardial oxygenation, invasive measures of blood oxygen and coronary blood flow within and beyond the boundaries of the coronary autoregulation zone.

## Materials and Methods

### Ethical Statement

This study was approved by the Veterinary Services at the Department of Agriculture and Nature of the Canton Bern, Switzerland [#BE 103/14] (7). The study was carried out in accordance with national and local animal care regulations and adheres to the ARRIVE guidelines.

### Experimental Anesthesia Surgical Protocol

**Figure 1:**
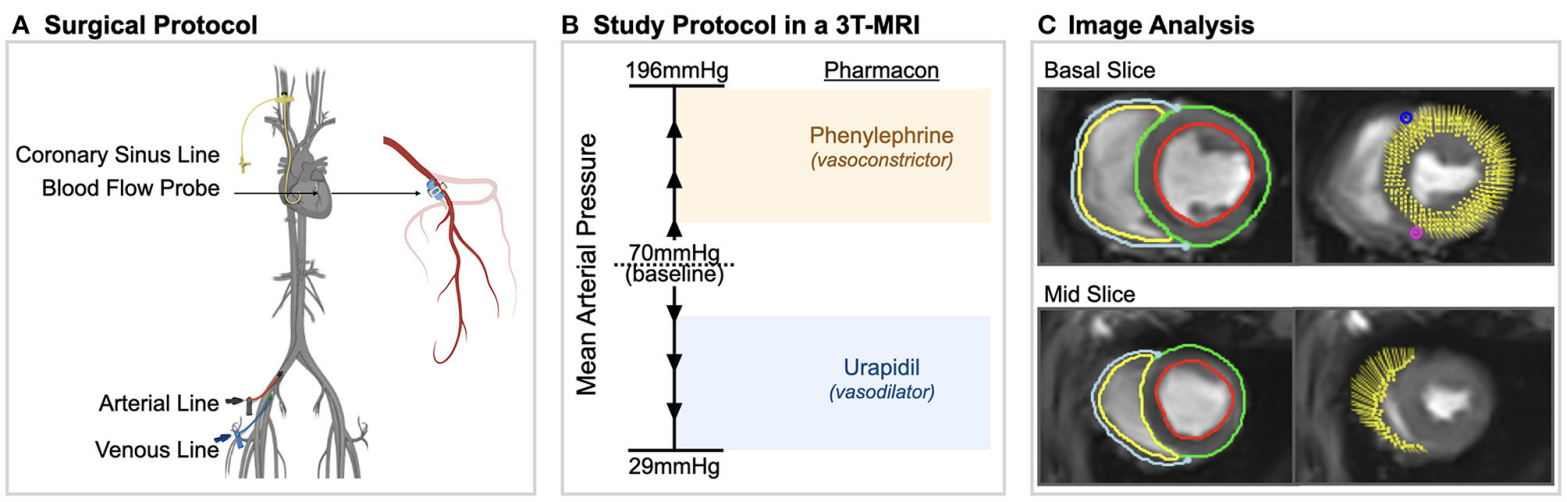
Experimental procedures. **(A)** Surgical procedures included placement of arterial, venous and coronary sinus lines and a blood flow probe on the left anterior descending coronary artery in anesthetized swine. **(B)** In a 3 tesla (T) magnetic resonance imaging (MRI) machine, mean arterial blood pressure was varied in steps of 10–15 mmHg from 29 to 196 mmHg using phenylephrine and urapidil. At each level, oxygenation-sensitive (OS) cine images along with invasive measurements were acquired. **(C)** Using CMR feature tracking-software, left and right ventricular systolic and diastolic strain measurements were obtained from the same cine images in which myocardial oxygenation was measured.

For acclimatization, the animals were housed at the University Veterinary Facilities for 48 h prior to the experiments. Fifteen swine (German Large White) were pre-medicated with 20 mg/kg ketamine and 2 mg/kg intra-muscular xylazine. The animals were induced with 10 mg of midazolam and 1 mg atropine and intubated. The animals were ventilated with a tidal volume of 6–8 ml/kg with a positive end-expiratory pressure of 5 mbar. Normocapnic end-tidal CO_2_-partial pressures of 35–40 mmHg were targeted. A venous line was placed in the femoral vein and anesthesia was maintained with continuous intra-venous fentanyl (5–30 μg/kg/h) as well as propofol (4–8 mg/kg/h) as required. 5'000 IU of heparin for prophylaxis of thrombosis as well as 75 mg of amiodarone for antiarrhythmic prophylaxis was administered. The femoral artery was cannulated for continuous invasive blood pressure measurements as well as intermittent blood gas analysis. Via access through the right internal jugular vein, an indwelling catheter was placed in the coronary sinus under surgical control. This was used to take blood gas measurements for determining myocardial oxygen extraction and derived invasive oxygenation measures. Afterwards a left-sided thoracotomy was performed and a perivascular flow probe (Transonic Systems, Ithica, NY, USA) was surgically attached to the proximal left anterior descending (LAD) coronary artery (Figure 1A).

### Pharmacological Manipulation of Blood Pressure

Inside of a clinical 3T magnetic resonance imaging scanner (MAGNETOM TRIO, Siemens Healthineers, Erlangen, Germany), MAP was adjusted in steps of 10–15 mmHg from a baseline level of 70 mmHg using urapidil and phenylephrine (Figure 1B), as published by our group previously (7). At each MAP-level, blood gas samples from the coronary sinus and the femoral artery were obtained, along with OS-CMR images. Phenylephrine is an α1-receptor agonist, constricting peripheral blood vessels and thereby increasing blood pressure. It has no effect on the β1-receptor and thus no impact on inotropy or chronotropy. Also, in contrast to other vasopressors like norepinephrine, it has minimal effect on vasomotor function of the coronary arteries (12). Phenylephrine was administered by infusion pump at a rate of 16–660 μg/min. Urapidil is a selective α1-receptor antagonist and is used to lower blood pressure by dilating peripheral blood vessels (13). Due to the longer half-life time, repetitive i.v.-doses of 5–10 mg were used. Throughout the study, arterial blood pressure, heart rate and coronary blood flow of the LAD were recorded continuously at a rate of one measurement per second.

### Experimental Imaging and Analysis

Oxygenation-sensitive (OS) cines were acquired in two short axis planes at base and mid-ventricle using a triggered balanced steady-state free precession sequence as reported before (7). Images were obtained in short end-expiratory apnea, with an acquisition time of four heartbeats per slice. Assessment of the myocardial oxygenation response was measured in the end-systolic frame of the OS-CMR images for each MAP level as reported before (14, 15). The signal measured in the left myocardium was reported as relative change in comparison to the baseline level of 70 mmHg. By assessors blinded to previous findings of myocardial oxygenation and invasive measurements, myocardial deformation was analyzed on the same OS-CMR cines using feature tracking (FT) software. Epicardial as well as endocardial contours were manually traced for the left ventricle and right ventricular free wall on both short axis slices at end-diastole (Figure 1C). Strain was assessed for the circumferential orientation. For systolic parameters, peak global circumferential strain (GCS), time to peak strain (TTP) and systolic strain rate (sSR) were extrapolated from the analysis. Early diastolic strain rate (dSR) was used as a measure of diastolic function. End-diastolic and systolic area of the chamber lumen was measured at the mid-ventricular short-axis plane, and fractional area change was calculated. All image analysis was performed with CVI^42^ (CircleCVI Inc., version 5.10, Calgary, Canada).

### Statistical Analysis

**Figure 2:**
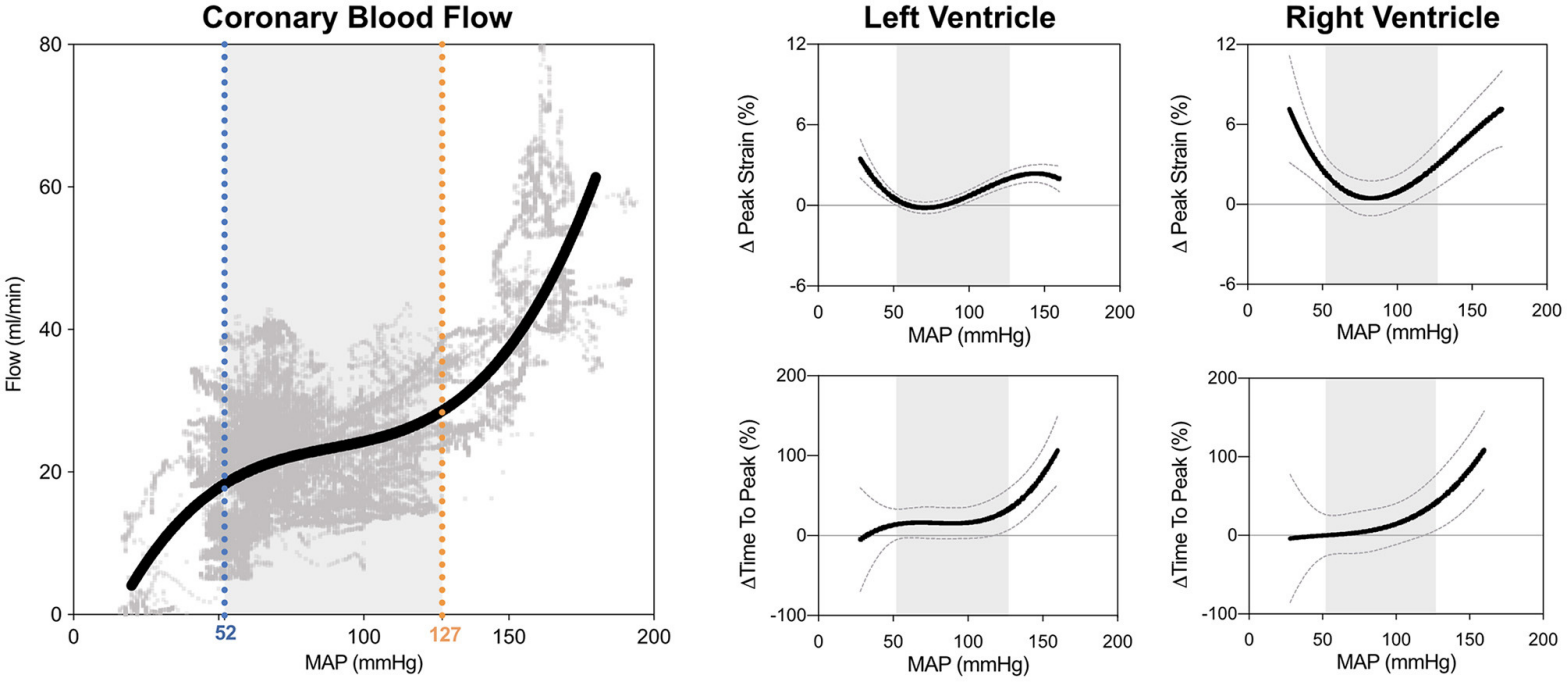
Biventricular systolic strain measures in relation to blood pressure. The limits of the coronary autoregulation zone (ARZ) as calculated from invasive flow measurements are shown on the left. Non-linear relationships and 95% confidence intervals of the fit are shown between mean arterial blood pressure (MAP) and peak circumferential strain and time to peak strain for the left ventricle (middle) and right ventricle (right). Gray shading depicts the ARZ.

For every subject the FT data were compared to the individual baseline-values (70 mmHg). The autoregulation zone (ARZ) was determined from the coronary blood flow data, from which a non-linear regression curve accounting for repeated measurements per subject was generated from 55'368 data points. The limits of the autoregulation zone were calculated from the first and second derivative of the flow curve, defined previously by Guensch et al. (7). As shown in Figure 2, 52 mmHg (blue) was defined here as the lower limit of the autoregulation zone and 127 mmHg (orange) as the upper limit in this sample of anesthetized swine.

Initially, the linear relationship between continuous CMR-FT variables to different parameters of flow and oxygenation was assessed using R Studio (RStudio Inc., Boston, United States) with mixed linear models fitted with the *lmer* function in R (*lme4* package) accounting for multiple measurements per animal by including subject identification as a random intercept. To assess the categorical differences in CMR-FT and invasive measures inside and outside of the ARZ, data at each level was categorized into three groups, levels below the ARZ (<52 mmHg), levels within the ARZ (52–127 mmHg), and a third group of levels above the ARZ (>127 mmHg). These groups were compared with a mixed effects model and the *emmeans* package (16) accounting for repeated measures per animal. For visualization purposes, CMR-FT measurements were compared to MAP, and other invasive measures using polynomial non-linear regression. Statistical significance was defined with a two-sided *p*-value of <0.05. GraphPad Prism version 9.0 (GraphPad Software, La Jolla California USA), *R* software (version 3.5.0, R Foundation for Statistical Computing, Vienna, Austria) were used for analysis.

## Results

Mean arterial blood (MAP) pressure in anesthetized swine was manipulated through a range of 29–196 mmHg from a baseline blood pressure of 70 mmHg. In total, 105 levels were targeted from 10 different subjects, at which CMR imaging, and invasive measures of CBF and blood oxygenation were acquired. Five of the fifteen animals were excluded prior to analysis: two suffered ventricular fibrillation during surgery; one had underlying peri-myocarditis that was discovered during surgery; there were experimental hardware problems with the remaining two. FT could be performed on 99 of the levels (94%), with 6 levels excluded due to gating issues.

### FT-CMR in the Autoregulation Zone

As can be seen in Figure 2, GCS of both ventricles was attenuated both above and below the coronary ARZ thresholds. This yielded a u-shaped non-linear relationship (*p* < 0.05) where a more positive value represented poorer strain, which was confirmed with categorical assessments (Table 1). In comparison to a baseline MAP of 70 mmHg GCS was compromised by 1.2 ± 0.4% at hypotensive levels below 52 mmHg (*p* = 0.041 vs. ARZ), and also attenuated by 2.1 ± 0.4% when MAP was higher than 127 mmHg (*p* < 0.001 vs. ARZ). This was even more pronounced in the RV, with an attenuation in GCS of 4.4 ± 1.2% below the ARZ (*p* = 0.004 vs. ARZ), and an attenuation of 5.4 ± 1.4% above the ARZ (*p* < 0.001). On the other hand, time to peak strain (TTP) increased only above the upper limit with a prolongation of 52 ± 21 ms for the LV (*p* = 0.008 vs. ARZ) and 67 ± 30 ms for the RV. This trend was matched by a reduction in systolic strain rate above the upper ARZ limit, whereas diastolic strain rate was not associated to mean arterial blood pressure. When assessing the end-diastolic area of the mid-ventricle, a larger ventricular lumen area was observed for both the LV and the RV above the ARZ in comparison to area at levels within the ARZ, while hypotension below the ARZ only resulted in a small but significant decrease in RV area in the short axis images with no impact on the LV.

**Table 1:** ANOVA comparisons for imaging and invasive measures below and above the autoregulation zone.

	**Below ARZ, <52 mmHg**	**P vs. ARZ**	**Within ARZ, 52–127 mmHg**	**Above ARZ, >127 mmHg**	**P vs. ARZ**
**LEFT VENTRICLE**					
ΔPeak strain, GCS (%)	1.2(0.4)	0.041^*^	0.4(0.2)	2.1(0.4)	<0.001^*^
ΔTime to peak, TTP (ms)	20(21)	0.920	19(19)	52(21)	0.008^*^
ΔSystolic strain rate, sSR (/s)	0.02(0.14)	0.116	−0.13(0.12)	0.08(0.15)	0.042^*^
ΔDiastolic strain rate, dSR (/s)	−0.02(0.14)	0.723	−0.08(0.08)	−0.13(0.16)	0.183
ΔEnd-diastolic area (mm^2^)	−0.04(3.8)	0.623	1.5(3.0)	17.8(4.0)	<0.001^*^
ΔEnd-systolic area (mm^2^)	6.4(2.9)	0.740	5.4(2.0)	27.0(30.7)	<0.001^*^
ΔFractional area change (%)	−15.4(6.0)	0.046^*^	−6.8(5.1)	−15.9(6.2)	0.043^*^
**RIGHT VENTRICLE**					
ΔPeak strain, GCS (%)	4.4(1.2)	0.004^*^	1.0(0.9)	5.4(1.4)	<0.001^*^
ΔTime to peak, TTP (ms)	4(29)	0.596	14(25)	67(30)	0.011^*^
ΔSystolic strain rate, sSR (/s)	0.13(0.13)	0.400	0.02(0.08)	0.70(0.15)	<0.001^*^
ΔDiastolic strain rate, dSR (/s)	−0.16(0.19)	0.903	−0.18(0.13)	−0.29(0.21)	0.568
ΔEnd-diastolic area (mm^2^)	−5.0(3.8)	0.022^*^	2.5(2.9)	4.6(4.1)	0.029^*^
ΔEnd-systolic area (mm^2^)	0.1(4.8)	0.995	0.1(4.1)	16.3(5.1)	<0.001^*^
ΔFractional area change (%)	−6.0(3.7)	0.219	−2.1(2.8)	−24.5(4.0)	<0.001^*^
**OXYGEN MEASURES (LEFT VENTRICLE) AND HEART RATE**					
ΔLAD CBF blood flow (ml/min)	−20.8(8.3)	<0.001^*^	9.9(6.7)	45.5(10.5)	<0.001^*^
Myocardial oxygenation, OS-CMR (%)	−0.8(0.9)	0.015^*^	1.3(0.6)	2.0(1.0)	0.431
ΔCoronary sinus saturation, ScsO_2_ (%)	−6.9(2.5)	<0.001^*^	2.8(2.0)	2.0(3.1)	0.762
ΔOxygen extraction ratio, O_2_er (%)	6.3(2.0)	<0.001^*^	−3.6(1.3)	−4.6(2.7)	0.731
ΔΩ, Oxygen excess	−0.17(0.06)	<0.001^*^	0.12(0.04)	0.14(0.08)	0.750
ΔHeart rate (beats per minute)	−2.4(3.2)	0.348	0.5(2.3)	−1.5(3.9)	0.594

*Mean (standard error) change from the baseline level (70 mmHg) acquired from a mixed effects model are shown measurements at levels below, inside and above the autoregulation zone (ARZ) as determined by the invasive measures of coronary blood flow (CBF) in the left anterior descending coronary artery (LAD)*.

* *p < 0.05 represents a significant difference in comparison to values inside the autoregulation zone (52–127 mmHg)*.

### Relationship of LV Feature Tracking to Measures of Oxygenation and Blood Flow

**Figure 3:**
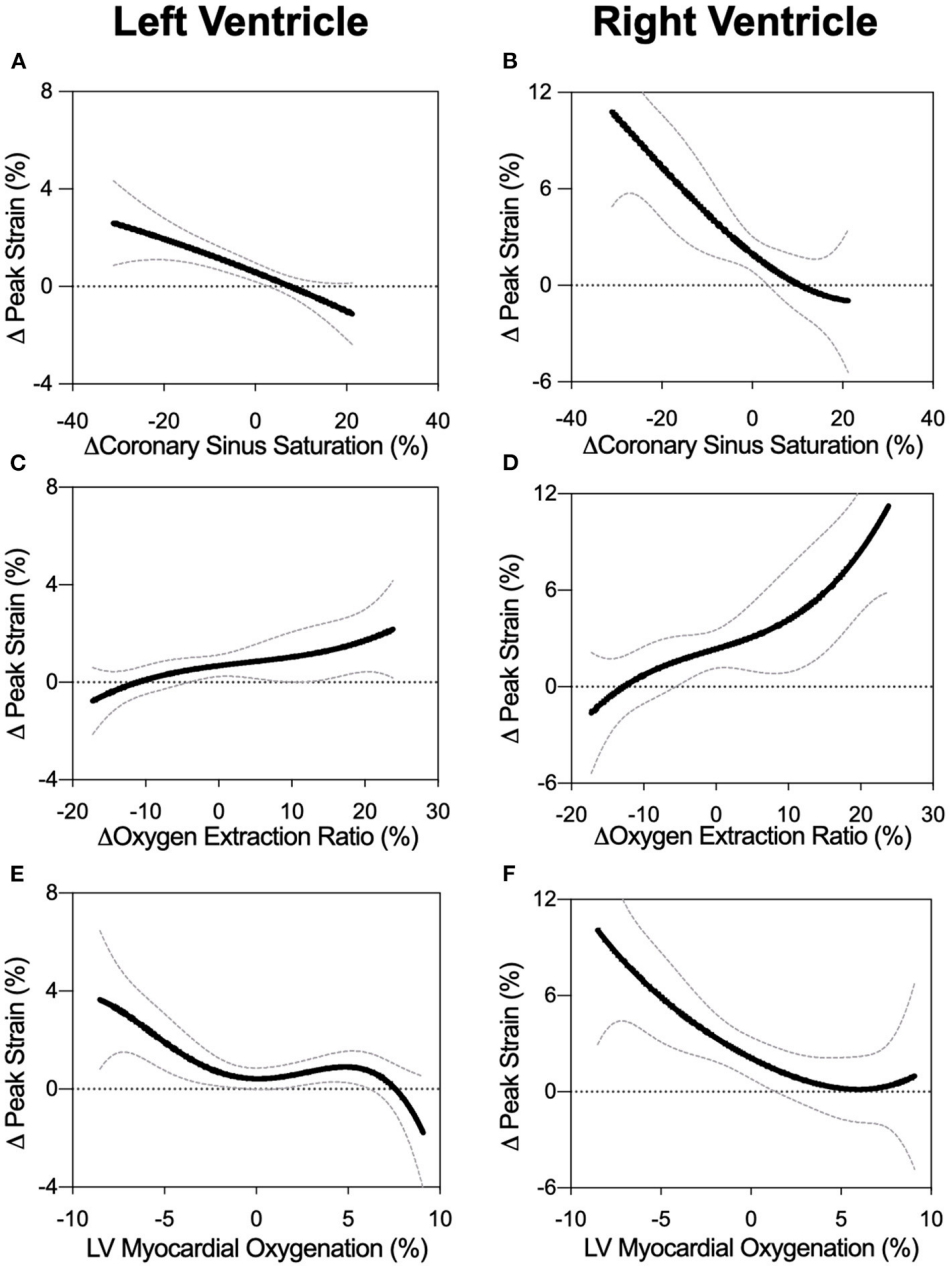
Biventricular GCS in relation to measures of oxygen. Non-linear fits are shown for left and right ventricular peak strain in comparison to changes in coronary sinus oxygen saturation (ScsO_2_, panels **A** and **B**), oxygen extraction ratio (O_2_er, panels **C** and **D**), and the myocardial oxygenation response of the left ventricular myocardium (OS-CMR, panels **E** and **F**).

As observed in Figure 3E, LV-GCS as a marker of systolic function showed a non-linear relationship to myocardial oxygenation measured by OS-CMR. In this non-linear regression analysis, an attenuation in GCS was observed with decreasing oxygenation in OS-CMR (<0%). This is supported with the linear analysis shown in Table 2 and Supplementary Figure 1. When investigating specifically MAP levels below the autoregulation zone where myocardial deoxygenation occurs (Table 1), a linear relationship was observed between GCS and OS-CMR (β = 0.216, *p* = 0.036, Table 2), indicating strain dropped by 0.216% with each drop in 1% of myocardial oxygenation. On the other hand, at MAP above the ARZ no linear relationship was observed.

**Table 2:** Association of FT and oxygenation measurements outside the autoregulation zone.

	**ΔLAD CBF**		**OS-CMR**		**ΔScsO**_**2**_		**ΔO**_**2**_ **er**	
	**β**	***P***	**β**	***P***	**β**	***P***	**β**	***P***
**All MAP LEVELS (29–196 mmHg)**								
Left ventricle								
ΔPeak strain, GCS (%)	−0.001	0.869	−0.083	0.109	−0.069	<0.001^*^	0.058	0.006^*^
ΔTime to peak, TTP (ms)	−0.077	0.564	2.136	0.143	−0.503	0.156	0.536	0.169
ΔSystolic strain rate, sSR (/s)	−0.001	0.421	−0.009	0.467	−0.006	0.006^*^	0.005	0.026^*^
ΔDiastolic strain rate, dSR (/s)	−0.001	0.347	0.015	0.146	−0.004	0.588	0.005	0.548
Right ventricle								
ΔPeak strain, GCS (%)	−0.043	0.008^*^	−0.425	0.002^*^	−0.243	<0.001^*^	0.212	0.001^*^
ΔTime to peak, TTP (ms)	−0.101	0.647	0.687	0.759	−1.123	0.121	1.362	0.081
ΔSystolic strain rate, sSR (/s)	−0.003	0.012^*^	−0.038	0.023^*^	−0.013	0.006^*^	0.017	0.001^*^
ΔDiastolic strain rate, dSR (/s)	−0.001	0.856	0.048	<0.001^*^	−0.006	0.529	0.007	0.511
**BELOW ARZ (<52 mmHg)**								
Left ventricle								
ΔPeak strain, GCS (%)	−0.028	0.047^*^	−0.216	0.036^*^	−0.059	0.258	0.035	0.480
ΔTime to peak, TTP (ms)	0.472	0.204	2.203	0.382	−1.367	0.113	1.116	0.183
ΔSystolic strain rate, sSR (/s)	−0.004	0.017^*^	−0.019	0.063	−0.007	0.128	0.003	0.436
ΔDiastolic strain rate, dSR (/s)	0.026	0.002^*^	0.037	0.072	0.004	0.738	0.017	0.106
Right ventricle								
ΔPeak strain, GCS (%)	−0.107	0.071	−0.145	0.662	−0.330	0.156	0.301	0.079
ΔTime to peak, TTP (ms)	0.155	0.719	−1.046	0.589	−1.051	0.355	0.143	0.856
ΔSystolic strain rate, sSR (/s)	−0.010	0.007^*^	−0.048	0.010^*^	−0.019	0.122	0.019	0.078
ΔDiastolic strain rate, dSR (/s)	0.004	0.102	0.014	0.350	0.007	0.215	-0.008	0.268
**ABOVE ARZ (>127 mmHg)**								
Left ventricle								
ΔPeak Strain, GCS (%)	−0.016	0.180	0.018	0.894	−0.046	0.641	0.103	0.345
ΔTime to peak, TTP (ms)	−2.050	0.454	7.601	0.149	3.216	0.011^*^	-2.630	0.138
ΔSystolic strain rate, sSR (/s)	−0.001	0.936	−0.050	0.360	0.008	0.299	-0.006	0.504
ΔDiastolic strain rate, dSR (/s)	0.001	0.977	0.045	0.163	−0.015	0.551	0.005	0.857
Right ventricle								
ΔPeak strain, GCS(%)	−0.013	0.868	−0.700	0.146	0.969	0.542	1.185	0.230
ΔTime to peak, TTP (ms)	−2.047	0.256	−3.341	0.520	28.36	0.106	10.36	0.521
ΔSystolic strain rate, sSR (/s)	−0.003	0.568	−0.127	0.092	−0.043	0.642	0.162	0.028^*^
ΔDiastolic strain rate, dSR (/s)	0.010	0.147	0.073	0.104	−0.139	0.114	0.123	0.076

*Parameter coefficients (β) and corresponding p-values represent linear associations between strain and measures of oxygenation from all MAP levels below the autoregulation zone (ARZ: <52 mmHg), or independently assessed above the ARZ (>127 mmHg). CBF, coronary blood flow of the left anterior descending coronary artery (LAD); O_2_er, oxygen extraction ratio; OS-CMR, oxygenation-sensitive cardiovascular magnetic resonance; ScsO_2_, coronary sinus oxygen saturation*.

* *p < 0.05*.

Similar relationships were observed for CBF, where only below the autoregulation zone did a decrease in CBF relate to attenuated FT systolic (GCS: β = −0.028, *p* = 0.047, sSR: β = −0.004, *p* = 0.017) and diastolic measures (dSR: β = 0.026, *p* = 0.002), but no association was observed between strain and CBF at high MAP.

Invasive blood measures of coronary sinus oxygen saturation (ScsO_2_) and oxygen extraction ratio (O_2_er) were linearly correlated to GCS across the entire MAP range (Figures 3A,C). This was also observed with systolic strain rate, while there was no link to diastolic strain rate for any MAP range (Table 2).

### Relationship of RV Feature Tracking to Measures of Oxygenation and Blood Flow

RV strain measurements showed multiple associations with CBF of the LAD and oxygen measures when assessing the entire MAP range (Figures 3B,D,F). In particular for OS-CMR, a reduction in left ventricular myocardial oxygenation was associated with a reduction in RV systolic (GCS: β = −0.425, *p* = 0.002, sSR: β = −0.038, *p* = 0.023) and diastolic parameters (dSR: 0.048, *p* = 0.001). In line with the LV, RV-GCS and sSR improved with a greater coronary sinus saturation and reduced oxygen extraction ratio (O_2_er). These systolic strain measures also improved with enhanced CBF, while none of the invasive measures were correlated with RV diastolic strain rate. In contrast to conditions in the LV, these associations between FT and CBF and oxygen measures were not observed when investigating only MAP levels below the ARZ.

## Discussion

We demonstrate in an anesthetized swine model that biventricular systolic function was compromised at blood pressures outside the coronary autoregulation zone. Left ventricular circumferential peak strain (GCS) was attenuated in a non-linear relationship with decreasing myocardial oxygenation in OS-CMR cine images. Specifically, below the autoregulation zone (ARZ), LV-GCS compromise was associated in linear fashion with myocardial deoxygenation, invasive measures of blood deoxygenation and reduced CBF in the LAD.

### Advantages of Non-invasive Imaging

Many of the reported markers for investigating adverse effects of blood pressure depressions and elevations do not convey their immediate risk to the patient, nor suggest corrective measures. These include blood biomarkers such as the troponins, which take a few hours to rise, markers of other organ injury, or post-surgical morbidity and mortality. By the time such abnormalities develop, it is usually too late for causative treatment. Functional imaging is advantageous in this regard as it can usually be non-invasively acquired within a short time period in a point-of-care setting. In this study we utilized CMR cine images, as information on myocardial oxygenation and function can be simultaneously investigated and linked. OS-CMR sequences use the blood oxygen level-dependent (BOLD) effect to delineate changes in the myocardial oxygen supply and demand balance, avoiding the requirement of contrast agents.

Multiple studies investigating myocardial oxygenation responses in healthy animal models as well as in various disease models (7, 14, 15, 17–21) have emerged over the last decade. As we use a cine variant, it measures a cardiac phase every 40 ms, thus strain can be analyzed from the same set of images. Other studies were also able to show associations between resting myocardial oxygenation and strain parameters (14). In order to look at subtle myocardial function changes, intraoperative echocardiography provides an accessible measure to measure strain (22), albeit without the benefit of tissue oxygenation.

### Clinical Implications of LV Strain at Hypotensive Periods

A mildly lowered blood pressure can be beneficial as it is easier for the ventricle to unload when there is less systemic resistance. However, afterload reduction, as during anesthesia, is only beneficial to a point where diastolic myocardial or other organ perfusion pressures become critically low. Peri-operative hypotension is a common occurrence, and is associated with a greater risk of peri-operative myocardial infarction (23). Especially elderly high-risk patients are prone to developing hypotension and myocardial ischemia. In humans, analysis from a database of 33,000 non-cardiac surgeries reported that myocardial injury and acute kidney injury are noted after even short periods of intraoperative MAP under 55 mmHg (24), a lower limit similar to that of our anesthetized swine. Monitoring hypotension is important intra- as well as post-operatively. It has been shown that after moderate- to high-risk non-cardiac surgery, eight percent of patients experienced two cumulative hours of MAP-levels below 60 mmHg, and that this hypotension was associated with troponin-defined myocardial injury after non-cardiac surgery (MINS) (25). According to coronary blood flow data of this study, this lower coronary autoregulatory limit was at a MAP of around 52 mmHg in anesthetized swine. A likely rationale for the reduction in strain at low blood pressure levels is decreased perfusion and subsequent tissue oxygenation below the lower autoregulatory limit (7). At low oxygenation the myocardium suffers lack of substrates and can go into a short-term state of hibernation. It reduces workload to adjust to reduced oxygen supply, which results in attenuated strain (26). This can be observed by the linear association between invasive measurements and function at MAP levels below the ARZ.

We, as well as others, observed that lower autoregulatory limits differ on an individual level as well. Consequently, intraoperative measures of strain, through using intraoperative echocardiography, may guide anesthesiologists along individualized blood pressure management corridors. Beyond cardiac protection, other organs will benefit from adequate perfusion pressure management, too. For instance, ischemic symptoms of the central nervous system can arise at lower MAP limits of 45–55 mmHg in supine subjects (27). Importantly, there is data for cerebrovascular autoregulation that the lower limit of the cortical ARZ may be as high as 90 mmHg in some individuals (28). Thus, it may also be inferred that autoregulation may be compromised in patients with pre-existing coronary pathologies. Since the individual and organ-specific ARZ of every patient are, in effect, a black box, functional measurements would be advantageous to target a MAP that ensures adequate organ function. Therefore, intraoperative patient-side myocardial strain analysis may be a suitable tool. While other vital organs also have their own autoregulation, the resultant perfusion pressure is still driven in part by proper left ventricular function. Therefore, it appears vital to monitor cardiac function when managing blood pressure around a low MAP threshold.

### Clinical Implications of LV Strain at Hypertensive Periods

**Figure 4:**
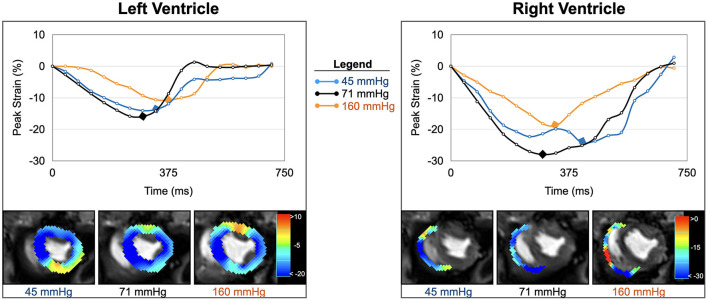
Strain inside and outside the autoregulation zone. Circumferential strain curves and strain overlays at end-systole of one animal are shown for the left and right ventricle. In comparison to baseline (black, 71 mmHg), biventricular strain at low blood pressure (blue, 45 mmHg), and high blood pressure (orange, 161 mmHg) both demonstrate attenuations in peak strain (yellow-red- color in overlay) and prolongations in time to peak strain (marked by diamond in curve).

Our animal data indicate that hypertension did not limit myocardial perfusion and oxygenation. Nevertheless, biventricular function was compromised and there was end-diastolic dilation of both ventricles. Not only is systolic function limited, but systolic contraction also becomes prolonged as seen by time to peak strain, although there is no change in heart rate (Figure 4). One reason for this might be that the increased workload to counteract high blood pressures compromises function mechanically despite abundant tissue oxygenation. In a clinical context, this may be the case in acute increases during a hypertensive crisis, leading to left-ventricular systolic dysfunction (29). Intraoperative hypertension and tachycardia are associated independently with adverse outcomes after long non-cardiac surgeries, regardless of underlying medical conditions (30). Other studies were inconclusive about associations between intraoperative hypertension and post-surgical morbidity (2). Myocardial oxygenation did not appear compromised by hypertension in our healthy animal model, and since there was even excess oxygen delivery as seen at OS-CMR, blood oximetry and coronary blood flow (7), it may not be immediately clear why these healthy hearts should be vulnerable at hypertension. In fact, we observed a decoupling of myocardial strain from invasive measurements. The non-linear regression graph (Figure 2) indicates that systolic function starts to deteriorate at a MAP level, which is lower that the coronary blood flow-defined upper autoregulatory limit. This suggests that with progressive hypertension, myocardial function may be primarily affected by afterload. In particular, hypertensive strain dysfunction was significantly pronounced in the afterload-sensitive right ventricle.

### Clinical Importance of RV Strain

The RV must match the output of the LV. Peri-operative RV dysfunction not only leads to LV underfilling but can also cause upstream problems like venous organ congestion (31, 32). In the presence of chronic high RV afterload, such as in pulmonary hypertension, the RV will progressively adapt with wall hypertrophy (33). However, during short term afterload swings such as those seen in peri-operative situations, rapid adaptation is needed. The RV may dilate to maintain stroke volume (33), and we observed this in our animal model as well. However, concomitant reduction in contractile function can lead to RV failure and low cardiac output (33). A major difference when compared to the LV is the greater sensitivity of the RV to changes in afterload. Our data indicate that a rapid increase in afterload is poorly tolerated, however right ventricular and pulmonary invasive measurements were not acquired in our analysis to confirm this analysis. Also due to the high compliance of the RV in comparison to the LV, greater changes in strain were observed outside the ARZ.

### Strain as an Advantageous and Specific Marker for RV Function

Particularly with functional imaging modalities, the RV appears less well-researched than the LV. FT of the RV free wall offers functional monitoring. It signifies already deterioration before RVEF declines and is therefore of prognostic values (34).

While the OS cine modality is suitable for assessing the LV, the myocardial oxygenation response of the RV is not as reliably described with the sequence used. This is due to limitations of spatial resolution defined by the thin RV wall. An advantage of FT and functional analysis is therefore that the RV can be studied even when the target myocardium is thin. Although phenylephrine is reported to increase pulmonary arterial pressure (35), we cannot provide pertinent hemodynamic data in this study, although our RV strain results are highly suggestive in this regard. This study is limited in the fact we did not measure right ventricular or pulmonary hemodynamic measures. However, in a swine model with a surgically constricted pulmonary artery, it was reported that RV GCS measured by echocardiography was linearly correlated with RV systolic pressure, which indicates that free wall strain can aid in detecting acutely increasing RV afterload (36). Our oximetry data, i.e., ScsO_2_ and O_2_er, also reflect global myocardial oxygenation and are not specific to the RV, since the coronary sinus drains venous blood from both ventricles. RV venous drainage both into the coronary sinus and directly into the right atrial and RV cavum precludes any accurate measure of total RV oxygen consumption.

Although changes in RV systolic and diastolic parameters appeared to correlate linearly with many of LV-biased physiological measures it is also known that both RV perfusion and oxygen extraction have key characteristics that differ from the LV (37). The RV is known to have less effective pressure-flow autoregulation (37, 38). This may also explain why we observed closer linear association in the RV of invasive measures across the entire MAP range. This emphasizes the importance of assessing both LV and RV independently with strain analysis, since they react differently to blood pressure changes.

### Translating From an Animal Model and Limitations

Our study shows that repetitive assessments of myocardial strain within a single subject across a range of MAP at both hypotension and hypertension demonstrate that alteration of blood pressure is an independent variable driving oxygenation and functional changes. We used an animal model since such a protocol could not be conducted in humans. The advantage of the repetitive measures in an animal model, is that the setting can be highly controlled, however in a clinical setting the changes in myocardial strain are likely a multifactorial process that can also be impacted additional variables such as preload, intracellular calcium dynamics and other factors. This is supported by some of the lower-dependency statistical relationships between strain and invasive measures, indicating further variables may play a role as well. Another advantage of the animal model is that multiple invasive measurements can be obtained to investigate both the impact of blood and oxygen supply represented by CBF vs. and variables depicting the oxygen-supply demand balance. Although there is a physiological link between the two components, a dissociation between perfusion and oxygenation measurements can occur based on the tissue oxygen requirements (39) and consequently oxygenation measurements can be a more sensitive marker of myocardial ischemia. Moreover, as mentioned above, the invasive measures have limitations as well as they do not measure the same regions of the heart, as CBF is measured in the LAD, while coronary sinus oxygen measurements collect blood from a wider region of the heart. Thus, these direct comparisons between the two can be confounded if there is heterogeneity in perfusion and workload across the ventricles. This an advantage of imaging, as a pixelwise approach can be performed of the myocardium.

Although strain values appear consistent with those in human studies (15), further comparisons are needed between the FT analysis of OS-CMR cines and standard cine acquisitions. Due to the difficulty in maintaining stable circulation at extreme MAP levels over longer periods of time, rapid acquisition was needed and thus there were only two short-axis slices were obtained and longitudinal cines were not recorded. The assessment of longitudinal strain, however, would have been advantageous, first because the fiber orientation the RV is mainly in this direction and second in the LV the micro-vessels are located in the subendocardial myocardial layer. Thus, functional subendocardial deficits would represent a very early marker of myocardial ischemia due to increasing wall pressure. While human and porcine cardiac physiology are comparable in many aspects, there are limitations to this animal model as well. Therefore, findings of this study may not necessarily represent human responses under similar conditions, especially not in cardiovascular disease. The used anesthetics might also have influenced myocardial function. While fentanyl has scarce effects on the heart (40), propofol frequently induces hypotension and reduces preload (41). The results are therefore more applicable to scenarios during general anesthesia, whereas awake subjects may respond differently.

### Conclusion

Biventricular function interrogated by cardiovascular magnetic resonance feature-tracking (CMR-FT) is compromised at blood pressures outside of both the upper and lower limit of the coronary autoregulation zone. Ventricular dysfunction at very low blood pressure was shown to be associated both with coronary sinus oximetry data and non-invasive assessment of myocardial tissue oxygenation measured by OS-CMR. During induced hypertension beyond the upper autoregulatory limit, myocardial deformation appears primarily related to the increase in afterload. Further studies are required to understand the effects of acute blood pressure swings on simultaneous myocardial function assessments. Since there are still no consistent definitions of intraoperative hypotension and hypertension (2), myocardial deformation may have the potential to guide blood pressure management. Moreover, these findings are translatable to a clinical peri-operative setting as coronary autoregulation limits differ among individuals. Peri-operative strain assessments could be particularly useful for identifying these individualized thresholds by detecting early signs of myocardial dysfunction prior to the onset of severe clinical sequalae, especially in patients with cardiovascular disease who are more at risk for peri-operative ischemia.

## Data Availability Statement

The raw data supporting the conclusions of this article will be made available by the authors, without undue reservation.

## Ethics Statement

The animal study was reviewed and approved by the Veterinary Services at the Department of Agriculture and Nature of the Canton Bern, Switzerland (#BE 103/14). The study was carried out in accordance with national and local animal care regulations and adheres to the ARRIVE guidelines.

## Author Contributions

This study was conceptualized by DG, KF, AV, and BE. Data was acquired and analyzed by KF, MN, CJ, SH, BW, SPH, BJ, and KF, MN, and DG wrote the first draft. All authors critically reviewed and approved the final manuscript.

## Funding

This work was funded by institutional funds of the Department of Anaesthesiology and Pain Medicine at the Bern University Hospital, Inselspital, University of Bern and the Foundation for Research in Anaesthesiology and Intensive Care Medicine in Bern Switzerland.

## Conflict of Interest

The authors declare that the research was conducted in the absence of any commercial or financial relationships that could be construed as a potential conflict of interest.

## Publisher's Note

All claims expressed in this article are solely those of the authors and do not necessarily represent those of their affiliated organizations, or those of the publisher, the editors and the reviewers. Any product that may be evaluated in this article, or claim that may be made by its manufacturer, is not guaranteed or endorsed by the publisher.
