# Supplementary material for: Assessment of Myocardial Function During Blood Pressure Manipulations Using Feature Tracking Cardiovascular Magnetic Resonance
Source: Front Cardiovasc Med. 2021 Oct 12;8:743849. doi: 10.3389/fcvm.2021.743849 (PMC8545897; doi:10.3389/fcvm.2021.743849)
Supplement: Supplementary file 1 [file Data_Sheet_1.PDF]

Supplementary Material

Supplementary Figure 1. Scatterplots of Peak Strain

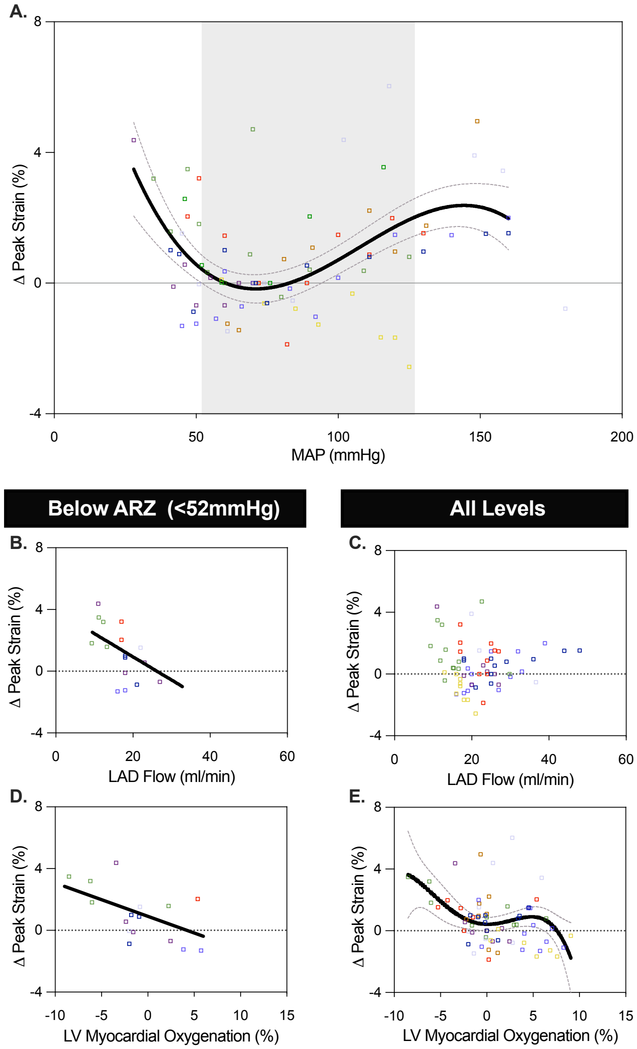

Scatterplots display the individual data points shown in Figures 2-3 and Table 2 of each acquired level (n=99). Each individual subject (n=10) is represented by a different color. A non-linear fit is shown for the change in peak strain for all levels (A), with grey depicting the autoregulatory zone (ARZ). The relationship for peak strain in comparison to blood flow of the left anterior descending (LAD) coronary artery is shown for levels with mean arterial pressure (MAP) below the ARZ (B), and for all levels across the blood pressure range (C). Similar trends are shown for the relationship between peak strain and myocardial oxygenation (D-E). For visualization of the linear trend below the ARZ (B,D) a univariate correlation line has been added to the graph.
